# Identification of key genes and pathways in chronic rhinosinusitis with nasal polyps and asthma comorbidity using bioinformatics approaches

**DOI:** 10.3389/fimmu.2022.941547

**Published:** 2022-08-17

**Authors:** Mingming Wang, Shuangmei Tang, Xiaoqi Yang, Xinyu Xie, Yang Luo, Shaojuan He, Xuezhong Li, Xin Feng

**Affiliations:** Department of Otorhinolaryngology, Qilu Hospital of Shandong University, National Health Commission (NHC) Key Laboratory of Otorhinolaryngology, Shandong University, Jinan, China

**Keywords:** chronic rhinosinusitis with nasal polyps, asthma, bioinformatic, hub genes, enrichment analysis

## Abstract

Patients with chronic rhinosinusitis with nasal polyps (CRSwNP) and asthma comorbidity (ACRSwNP) present severe symptoms and are more likely to relapse. However, the pathogenesis of ACRSwNP is not fully understood. The aim of this study was to explore the underlying pathogenesis of ACRSwNP using bioinformatics approaches. ACRSwNP-related differentially expressed genes (DEGs) were identified by the analysis of the GSE23552 dataset. The clusterProfiler R package was used to carry out functional and pathway enrichment analysis. A protein–protein interaction (PPI) network was built using the STRING database to explore key genes in the pathogenesis of ACRSwNP. The bioinformatics analysis results were verified through qRT-PCR. The Connectivity Map (CMap) database was used to predict potential drugs for the treatment of ACRSwNP. A total of 36 DEGs were identified, which were mainly enriched in terms of regulation of immune response and detection sensory perception of taste. Thirteen hub genes including AZGP1, AQP9, GAPT, PIP, and PRR4 were identified as potential hub genes in ACRSwNP from the PPI network. Analysis of the GSE41861 dataset showed that upregulation of CST1 in nasal mucosa was associated with asthma. qRT-PCR detection confirmed the bioinformatics analysis results. Tacrolimus and spaglumic acid were identified as potential drugs for the treatment of ACRSwNP from the CMap database. The findings of this study provide insights into the pathogenesis of ACRSwNP and may provide a basis for the discovery of effective therapeutic modalities for ACRSwNP.

## Introduction

Chronic rhinosinusitis (CRS) is a disease characterized by chronic inflammation of the nasal cavity and sinus mucosa. The prevalence of CRS in the United States is 12.5%, whereas its prevalence in European countries and in China is 7% to 27% and 8%, respectively, thus resulting in high economic and social burdens (1–3). CRS mainly presents as phenotypes, namely, CRS without nasal polyps (CRSsNP) and CRS with nasal polyps (CRSwNP), based on whether it is accompanied by polyps or not. CRSwNP patients present with severe symptoms, higher incidence of asthma, and higher recurrence rate after endoscopic nasal surgery compared with CRSsNP patients (4, 5). CRS and asthma result in significant health and economic burdens worldwide. Therefore, it is imperative to explore the pathogenesis of this chronic inflammatory disease, find key biomarkers, and elucidate the molecular basis to provide a basis for the development of new therapeutic strategies.

Previous studies on the relationship between CRS and asthma report numerous epidemiological and pathophysiological features linking these two diseases (6, 7). Incidence of asthma comorbidities varies widely in CRS patients, ranging from 7% to 71%. However, asthma comorbidities are highly correlated with the occurrence of nasal polyps (8). Asthma symptoms are more severe and challenging to treat in CRSwNP patients compared with CRSsNP subjects (9). Studies report that CRS is associated with poor asthma outcomes. Brinke et al. reported that 84% of adults with severe asthma showed mucosal abnormalities using sinus computed tomography (CT) scans and found a direct relationship between sinus mucosal thickness and bronchial inflammation in patients with severe asthma (10). Batra et al. explored the various comorbidities of CRS (asthma, inhalant allergy, and aspirin sensitivity), and the findings showed that asthma is the only independent predictor for the CRSwNP subtype (11). Pathophysiological mechanisms of asthma and CRSwNP are very similar. The two processes are driven by Th2-helper cell-mediated eosinophilic inflammation. Asthmatic patients exhibit a typical sinus inflammation pattern, with classic Th2 biomarkers in their nasal secretions, similar to CRS patients (12, 13). These findings result in a “unified airway” theory, suggesting that CRS is closely associated with the pathogenesis of asthma (14). The exact pathophysiological mechanism of CRSwNP comorbidity with asthma (ACRSwNP) has not been elucidated. Inflammatory characteristics and pathogenesis of ACRSwNP should be explored further.

Previous studies on the relationship between CRSwNP and asthma were all based on the nasal and bronchial tissues of the same CRSwNP patient, which may partially obscure the exact mechanism of asthma’s effect on the upper airway. To date, no study has compared the transcriptomic signature of the upper airway tissue of patients with CRSwNP and those with asthma. Therefore, the present study applied bioinformatics analysis to extensively explore potential genetic changes using transcriptomic data from ACRSwNP and asthma patients. This is the first study to simultaneously investigate transcriptomic data from the upper airways of patients with ACRSwNP and the upper and lower airways of asthmatics; this may provide insights into the mechanisms by which asthma affects the upper airways.

## Materials and methods

### Microarray dataset download

The GSE23552 dataset, the GSE41861 dataset, and the GSE104468 dataset were retrieved from the Gene Expression Omnibus (GEO) (https://www.ncbi.nlm.nih.gov/geo/) database (15). GSE23552 was obtained from the GPL5175 platform, Affymetrix Human Exon 1.0 ST Array (Affymetrix, Santa Clara, CA, USA). The GSE23552 dataset comprised microarray data of 10 inflamed nasal mucosa and 10 adjacent polyp samples from 10 ACRSwNP patients, and microarray data of 10 normal control nasal mucosa samples. Inflamed mucosa or nasal polyp was obtained from the anterior ethmoid cavity or middle meatus. The control group comprised normal tissues from the uncinate process or inferior turbinate. All patients with ACRSwNP met the diagnostic criteria for CRSwNP according to the European Position Paper on Rhinosinusitis and Nasal Polyps 2012 guidelines. In addition, patients were diagnosed with asthma by a physician, and presented with airway hyperresponsiveness or airway reversibility. Exclusion criteria for normal control patients included upper airway inflammation and immunodeficiency diseases. All subjects did not take intranasal and oral medications for ≥4 days prior to tissue donation. GSE41861 was obtained from GPL570 platform, Affymetrix Human Genome U133 Plus 2.0 Array (Affymetrix, Santa Clara, CA, USA). The dataset comprised microarray data of the nasal epithelial and bronchial epithelia brushings of 54 asthma patients and 30 healthy controls. The samples were obtained from the tissue bank at Translational Research Sciences, Hoffmann-La Roche, Nutley, New Jersey. GSE104468 was obtained from the GPL21185 platform, Agilent SurePrint G3 Human GE v3 8x60K Microarray (Agilent Technologies, Palo Alto, CA, USA). The dataset comprised microarray data of the nasal epithelial and bronchial epithelia tissues of 12 asthma patients and 12 normal controls. Details on each dataset are presented in **Table 1**.

**Table 1:** The details of GEO datasets.

GSE	PMID	Sample size (n)	Organization type	Platform
GSE23552	PMID: 20625511	ACRSwNP:10 Control:10	10 nasal polyps 10 Inflamed nasal mucosae 10 normal nasal mucosae	GPL5175
GSE104468 GSE41861	PMID: 28294656 PMID: NA	Asthma:12 Control:12 Asthma:54 Control:30	12 nasal epithelia 12 bronchial epithelia 12 nasal epithelia 12 bronchial epithelia 40 Nasal epithelial brushings 51 bronchial epithelial brushings 17 Nasal epithelial brushings 30 bronchial epithelial brushings	GPL21185 GPL570

GSE, GEO Series; PMID, PubMed Unique Identifier.

### Data processing and differential gene expression analysis

The corresponding platform annotation information file was retrieved from the GEO official website. R software (version 4.1.0; https://www.r-project.org/) was used to convert the probe names in the chip into the corresponding official gene symbols. Subsequently, the limma R package (version 3.48.3; https://www.r-project.org/) was used to normalize gene expression values using the quantile method (16). Inflamed nasal mucosa and polyp tissues of ACRSwNP were compared with normal nasal mucosa to identify the common differentially expressed genes (DEGs) between the two groups. Differential gene analysis was performed using the limma package, and Benjamini–Hochberg false discovery rate (FDR) was used to correct the *p*-value (17). The screening criteria used to obtain genes closely related to ACRSwNP were |log2| (fold change) > 2 and adjusted *p*-value < 0.05. The Venn Diagram Network tool (https://bioinfogp.cnb.csic.es/tools/venny/index.html) was used to identify DEGs common in the two groups, and a Venn diagram was generated. The base package in R software was used to generate heatmaps and volcano maps.

### Functional and pathway enrichment analysis

GO and KEGG pathway enrichment analysis was performed based on DEGs using the clusterProfiler package (version 4.0.5) in R software to explore the biological functions and related pathways of DEGs. A *p*-value < 0.05 and a *q*-value (adjusted *p*-value) < 0.05 indicated significantly enriched terms and pathways (18). Enrichment results were visualized using the ggplot2 R package (version 3.3.5).

### PPI network construction and identification of hub genes

The DEGs were uploaded to the STRING database (https://string-db.org/), which is a database for the prediction of protein–protein interactions (PPIs). The filter condition for the construction of a PPI network was a combined score > 0.2 (19). Cytoscape is an open-source network visualization and analysis software (20). Cytohubba is a plugin for Cytoscape used for the identification of central genes in the network (21). Four algorithms, namely, Degree, Density of Maximum Neighborhood Component (DMNC), Maximal Clique Centrality (MCC), and Clustering Coefficient, were used in Cytohubba plugin, to determine the top 20 hub genes (22, 23). A total of 13 hub genes were selected from the intersection of the results for each method. Furthermore, a subnetwork of the 13 hub genes from the PPI network was generated.

### Construction of the target gene–miRNA network and target gene–TF network

MiRNAs or TFs affect disease progression by regulating gene expression through interaction with target genes at the post-transcriptional level. NetworkAnalyst (https://www.networkanalyst.ca/) webserver (24), integrated TarBase miRNA database(http://microrna.gr/tarbase/), ENCODE transcription factor database(http://cistrome.org/BETA/), and RegNetwork database (http://regnetworkweb.org/) were used to predict the gene–miRNA–TF interaction network of the 13 hub genes. The network was visualized using Cytoscape software.

### Identification of key genes related to asthma in ACRSwNP

GSE41861 dataset comprised microarray data of the nasal epithelial and bronchial epithelia brushings of 54 asthma patients and 30 healthy controls. The dataset contains 57 nasal epithelial samples from participants with (*n* = 40) and without (*n* = 17) asthma and 81 samples from participants with (*n* = 51) and without (*n* = 30) asthma bronchial epithelial samples. The limma R package was used to analyze the GSE41861 dataset using the same criteria as GSE23552 [|log2| (fold change) >2 and adjusted *p*-value  < 0.05] to screen for DEGs in the nasal mucosal epithelium between asthmatics and healthy controls. For genes corresponding to multiple probes, the probe with the minimum value of |logFC| was taken as the expression level. The genes co-expressed in the nasal mucosa of asthma and ACRSwNP patients were screened by analyzing the DEG results obtained from the GSE41861 and GSE23552 datasets. Then, the co-expressed genes were searched in the GSE41861 gene expression matrix to explore the expression level of co-expressed genes in the bronchial epithelium of asthma patients and normal controls. These genes may be key genes in ACRSwNP that may be involved in the pathogenesis of asthma. Compared with CRSwNP, ACRSwNP had a higher level of upregulation of type 2 inflammation-related genes. Therefore, we searched the GSE23552 expression matrix to investigate the correlation of co-expressed genes with the mRNA expression levels of key cytokines and their receptors in type 2 inflammation, including IL5RA, IL5RB, IL4R, IL13RA1, and IL13RA2. The expression levels of co-expressed genes in the nasal polyps of ACRSwNP patients were verified by qRT-PCR, and the expression levels of co-expressed genes in the nasal epithelium and bronchial epithelium of asthma patients were verified by the dataset GSE104468.

### Patient recruitment

Samples were obtained from inpatients at Qilu Hospital of Shandong University. CRSwNP diagnosis was performed following the European Position Paper on Rhinosinusitis and Nasal Polyps (EPOS) 2012 (25). Nasal polyps of 30 ACRSwNP patients and 30 CRSwNP patients were collected as experimental samples. All CRSwNP patients with comorbid asthma had a history of asthma that was diagnosed by a respiratory specialist according to the Global Initiative for Asthma 2014 guidelines (GINA 2014). All ACRSwNP patients had no acute exacerbation of asthma symptoms within 3 months. For patients classified as CRSwNP, the previous asthma was first excluded by collecting the patient’s medical history, and then according to the diagnostic criteria of GINA2014, the patients were asked in detail whether they had one of the following symptoms to exclude current asthma: wheezing, shortness of breath, chest tightness, and cough. The inferior turbinate mucosa of 30 patients with deviated nasal septum and no sign of sinus inflammation at the CT scan was collected as the control samples. Patients with sinus inflammation were excluded after conducting CT scan of the sinuses. The patient had no history of nasal surgery, and had not used antibacterial drugs or glucocorticoid drugs within 1 month before surgery. All enrolled patients underwent chest CT examination to exclude bronchiectasis, chronic obstructive pneumonia, cystic fibrosis, and bronchitis. The exclusion criteria were as follows: history of immunodeficiency and other serious systemic diseases; history of hyperlipidemia and diabetes; history of bronchiectasis, chronic obstructive pneumonia, bronchitis, and pulmonary cystic fibrosis; and history of coronary heart disease and tumor. Differences in age and gender between the groups of patients were not statistically significant. This study was approved by the Medical Ethics Committee of Qilu Hospital of Shandong University. Demographic characteristics of subjects enrolled in this study are presented in **Table 2**.

**Table 2:** The demographic characteristics of all subjects.

	ACRSwNP (n = 30)	CRSwNP (n = 30)	Control (n = 30)	*p*-value
Age (years), mean ± SD	45.17 ± 13.5	42.97 ± 16.0	37.37 ± 12.7	0.09
Sex, male, *n* (%)	19 (63%)	25 (83%)	22 (73%)	0.22
History of smoking	7 (23%)	13 (43%)	8 (27%)	0.20
History of surgery	13 (43%)	11 (37%)	0 (0%)	0.60

ACRSwNP, chronic rhinosinusitis with nasal polyps comorbid with asthma; CRSwNP, chronic rhinosinusitis with nasal polyps; SD, standard deviation.

### RNA extraction and real-time quantitative PCR

Total RNA was extracted using TRizol Reagent (Sigma-Aldrich Co. LLC.) according to the manufacturer’s instructions. HiScript III RT SuperMix for qRT-PCR (Vazyme Biotech Co.) was used to synthesize complementary DNA from 1 μg of the RNA sample. β-actin gene was used as the internal control. qRT-PCR was performed using SYBR Green mix (Vazyme Biotech Co.) to evaluate gene expression levels. All qRT-PCR experiments were performed in duplicate. Relative gene expression levels were calculated using the standard 2−ΔΔCt method, followed by statistical analysis using GraphPad Prism 9.0 software. Mann–Whitney *U* test was used for comparison between the two groups as the data did not conform to normal distribution. Primers used in this study are listed in **Table 3**. qRT-PCR results have been uploaded to **Supplementary Table 1**.

**Table 3:** Sequences of primers.

Gene	Forward primer 5’-3’	Reverse primer 5’-3’
ALOX5AP	AAGTGGAGGACGAAAGCAGGAC	AGACCAGAGCACAGCGAGGAAA
AQP9	ACTGCTGATCGTGGGAGAAAA	GCGTTCGCCAGAGATAGATACG
AZGP1	AGGCCAGGGAGGACATCTTTTATG	AATGCTCCGCTGCTTCTGTTATTC
CCL13	AGCCAGATGCACTCAACGTC	TCTCCTTGCCCAGTTTGGTT
CST1	TGTGCCTTCCATGAACAGCCAG	CTGGCACAGATCCCTAGGATTC
EMR3	GATGGCTGCTTCCTGATACACG	CGTAGGTGATGACAGTCAGCAC
F13A1	CAACAGCCACAACCGTTACACC	CTTGGATCAGCACCGCCTCTTT
GAPT	TGGCACTGGAAACACCGTGTTG	CATGCCTTAAGCCAATGATGCGG
MUC7	TGCTTGCTTCTCGTTCAGTGA	GTGAGATTTGGGTGATTGGTGAT
NCF2	ACTACTGCCTGACTCTGTGGTG	CCTCCACTTGGCTGCCTTTCTT
PIP	CCTGCCTATGTGACGACAATCC	TCAGGGCAGATGCCTAATTCCC
PRR4	CCCGATTTCCTTCTGTCAGCCT	TGGCTTTCTGAAGGAAGTTATCTTC
STATH	TTTTGCGTAGAATTGGAAGATTCGG	AATCATGTCCTGCAGTTACTGATG

### Prediction of potential novel drugs for the treatment of ACRSwNP

The Connectivity Map (CMap) (https://portals.broadinstitute.org/cmap/) is a gene expression profiling database based on intervening gene expression to reveal functional links between small-molecule compounds, genes, and disease states. CMap compounds with negative enrichment scores are selected as potential drugs that can reverse the expression of DEGs (26). The CMap database was used to determine candidate small-molecule compounds that were highly negatively correlated with DEGs according to *p*-value < 0.05 and enrichment score < 0. In addition, potential therapeutic drugs that may reverse ACRSwNP gene expression were predicted. The top 10 small molecules were selected according to the enrichment score ranking.

## Results

### Identification of DEGs

**Figure 1:**
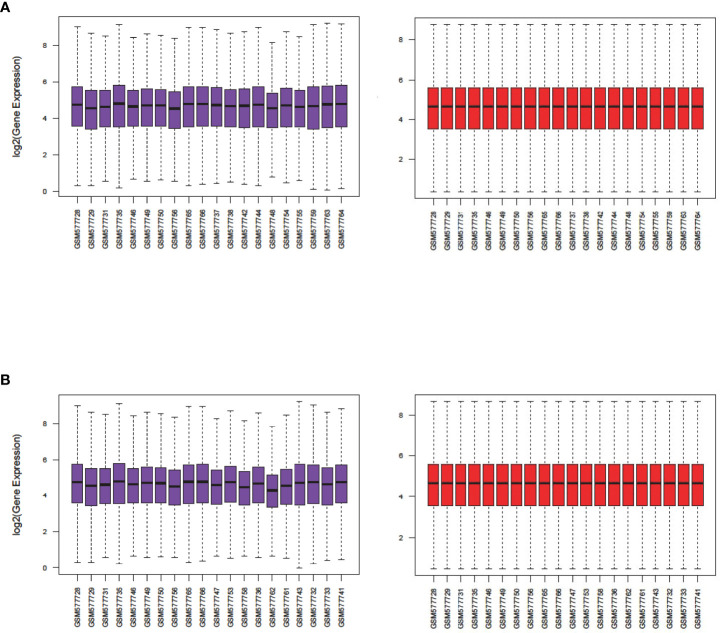
Boxplots of gene expression profiles of ACRSwNP samples in dataset GSE23552. **(A)** Normalization of the nasal polyp group, **(B)** normalization of the inflamed nasal mucosa group. The *x*-axis labels represent sample symbols, and the *y*-axis labels represent gene expression values. Purple and red bars represent data before and after normalization, respectively.

**Figure 2:**
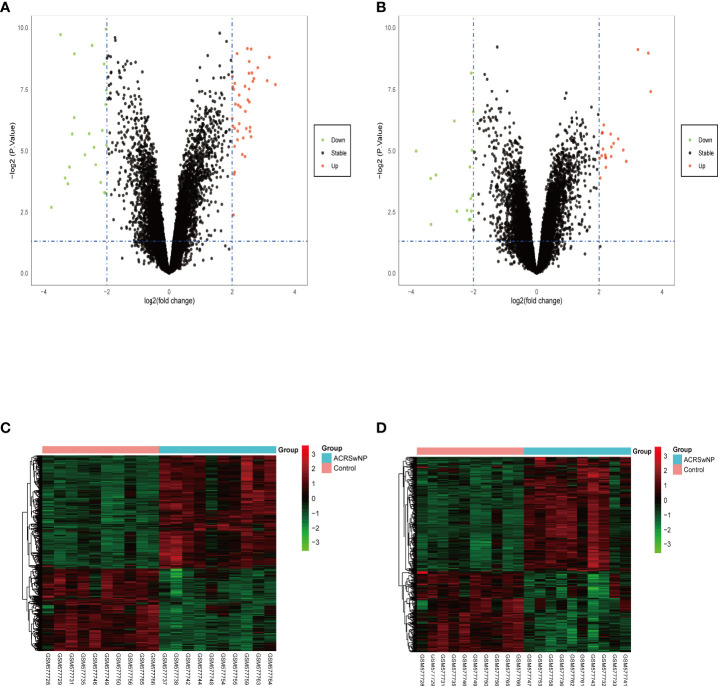
Volcano plots and heatmaps of the differentially expressed genes. **(A)** Volcano plots of the nasal polyp group. **(B)** Volcano plots of the inflamed nasal mucosa group. Red data points represent upregulated and green data points represent downregulated genes. The horizontal axis represents the fold change, and the vertical axis is the *p*-value. **(C)** Heatmap of DEGs in the nasal polyp group. **(D)** Heatmap of DEGs in the inflamed nasal mucosa group. The bar legend at the top right represents the log fold change in gene expression, with red representing an increase and green a decrease.

**Figure 3:**
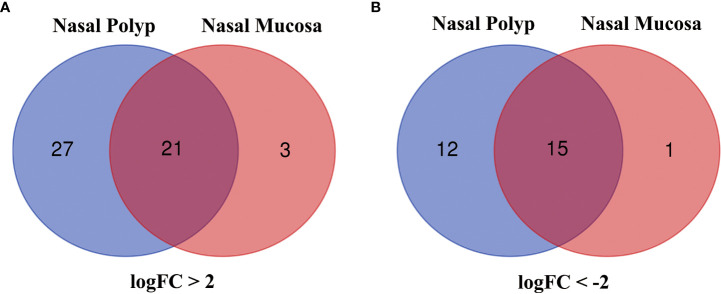
**(A)** Venn diagram of 21 DEGs upregulated in both groups, **(B)** Venn diagram of 15 DEGs downregulated in both groups. Blue represents the nasal polyp group and red represents the inflamed nasal mucosa group.

Analysis of GSE23552 to identify genes closely associated with ACRSwNP yielded 75 and 40 DEGs, respectively, in nasal polyps and inflamed nasal mucosa of ACRSwNP patients (**Figure 1**). Expression levels of DEGs were presented as volcano plots and heatmaps (**Figures 2A-D**). Venn diagram online tool was used to integrate genes expressed in nasal polyps and inflamed nasal mucosa (**Figures 3A, B**). A total of 36 genes were identified including 21 upregulated genes and 15 downregulated genes. The Venn diagram integrated DEGs are listed in **Table 4**.

**Table 4:** Venn diagram of the integrated DEGs in ACRSwNP.

DEGs	Gene names
Upregulated	AQP9 CDH26 CR1 CCL18 GAPT GPR97 CST1 SPP1 EMR1 HRH4 F13A1 VSTM1 ALOX5AP IL2RA POSTN CLC CST2 CCL13 NCF2 IL1RL1 EMR3
Downregulated	PIP SCN7A MSMB C6orf58 STATH GSTA2 CRISP3 PLN AZGP1 ACTG2 MYH11 MUC7 PRR4 LPO PRB4

DEGs, differentially expressed genes.

### GO and KEGG pathway enrichment analyses of DEGs

**Figure 4:**
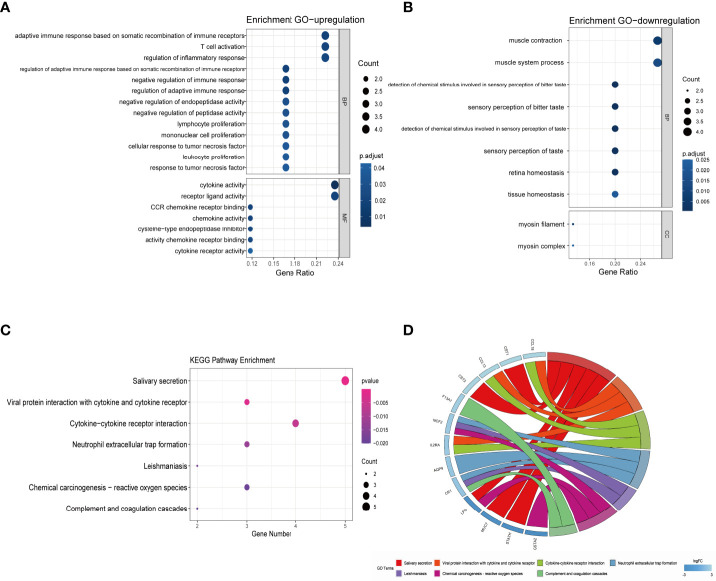
GO and KEGG pathway enrichment analysis of DEGs. **(A)** Bubble chart showing significantly enriched GO terms for upregulated DEGs in MF and BP terms. The horizontal axis represents the gene ratio, and the vertical axis represents the GO term. The size of the dots represents the number of enriched genes, and the shades of color represent *p*-values. **(B)** Bubble chart showing significantly enriched GO terms for downregulated DEGs in BP and CC terms. **(C)** Bubble chart showing enriched signaling pathways associated with DEGs. **(D)** A chord diagram showing distribution of DEGs in the KEGG pathway. The symbols for DEGs are presented on the left side of the graph, and their fold-change values are mapped by a color scale. Colored connecting lines represent involvement of genes in different KEGG pathways. GO, gene ontology; KEGG, Kyoto Encyclopedia of Genes and Genomes; DEG, differentially expressed gene; BP, Biological Processes; MF, Molecular Function; CC, Cellular Component; FC, fold change.

The results of functional enrichment and pathway analysis of DEGs are presented in **Table 5** and **Table 6**. GO terms are grouped into three categories, namely, Biological Processes (BP), Molecular Function (MF), and Cellular Component (CC). BP terms associated with the upregulated genes were adaptive immune response based on somatic recombination of immune receptors, T-cell activation, regulation of inflammatory response, negative regulation of immune response, and regulation of adaptive immune response. MF terms enriched for upregulated genes were cytokine activity, receptor ligand activity, CCR chemokine receptor binding, chemokine activity, chemokine receptor binding, cytokine receptor activity, and cysteine-type endopeptidase inhibitor activity (**Figure 4A**). Downregulated DEGs were significantly enriched in BP terms including muscle contraction, muscle system process, detection of chemical stimulus involved in sensory perception of bitter taste, sensory perception of bitter taste, detection of chemical stimulus involved in sensory perception of taste, and sensory perception of taste. Furthermore, myosin filament and myosin complex were the significantly enriched CC terms for downregulated genes (**Figure 4B**). Upregulated genes were not associated with enrichment of CC, and downregulated genes were not associated with MF terms owing to the small number of uploaded genes. KEGG pathway enrichment analysis indicated that DEGs were mainly enriched in salivary secretion, viral protein interaction with cytokine and cytokine receptor, cytokine–cytokine receptor interaction, and neutrophil extracellular trap formation pathways (**Figures 4C, D**).

**Table 5:** Results from GO enrichment analysis of DEGs.

Category	Term	Description	Gene count	Adjusted *p*-value	Gene ID
**Upregulated**					
BP	GO:0002460	Adaptive immune response based on somatic recombination of immune receptors built from immunoglobulin superfamily domains	4	1.85E−02	CR1/GAPT/CLC/IL1RL1
BP	GO:0042110	T-cell activation	4	2.02E−02	CDH26/CR1/IL2RA/CLC
BP	GO:0050727	Regulation of inflammatory response	4	2.15E−02	CR1/CCL18/IL2RA/IL1RL1
BP	GO:0002822	Regulation of adaptive immune response based on somatic recombination of immune receptors built from immunoglobulin superfamily domains	3	1.85E−02	CR1/CLC/IL1RL1
BP	GO:0050777	Negative regulation of immune response	3	1.85E−02	CR1/IL2RA/IL1RL1
BP	GO:0002819	Regulation of adaptive immune response	3	1.85E−02	CR1/CLC/IL1RL1
BP	GO:0010951	Negative regulation of endopeptidase activity	3	2.99E−02	CR1/CST1/CST2
BP	GO:0010466	Negative regulation of peptidase activity	3	3.02E−02	CR1/CST1/CST2
BP	GO:0046651	Lymphocyte proliferation	3	3.11E−02	GAPT/IL2RA/CLC
BP	GO:0032943	Mononuclear cell proliferation	3	3.11E−02	GAPT/IL2RA/CLC
BP	GO:0071356	Cellular response to tumor necrosis factor	3	3.58E−02	CCL18/POSTN/CCL13
BP	GO:0070661	Leukocyte proliferation	3	3.65E−02	GAPT/IL2RA/CLC
BP	GO:0034612	Response to tumor necrosis factor	3	3.74E−02	CCL18/POSTN/CCL13
MF	GO:0005125	Cytokine activity	4	3.90E−03	CCL18/SPP1/VSTM1/CCL13
MF	GO:0048018	Receptor ligand activity	4	1.99E−02	CCL18/SPP1/VSTM1/CCL13
MF	GO:0048020	CCR chemokine receptor binding	2	1.99E−02	CCL18/CCL13
MF	GO:0008009	Chemokine activity	2	1.99E−02	CCL18/CCL13
MF	GO:0004869	Cysteine-type endopeptidase inhibitor activity	2	2.23E−02	CST1/CST2
MF	GO:0042379	Chemokine receptor binding	2	2.40E−02	CCL18/CCL13
MF	GO:0004896	Cytokine receptor activity	2	4.29E−02	IL2RA/IL1RL1
**Downregulated**					
BP	GO:0006936	Muscle contraction	4	5.99E−03	SCN7A/PLN/ACTG2/MYH11
BP	GO:0003012	Muscle system process	4	1.36E−02	SCN7A/PLN/ACTG2/MYH11
BP	GO:0001580	Detection of chemical stimulus involved in sensory perception of bitter taste	3	5.08E−04	PIP/AZGP1/LPO
BP	GO:0050913	Sensory perception of bitter taste	3	5.08E−04	PIP/AZGP1/LPO
BP	GO:0050912	Detection of chemical stimulus involved in sensory perception of taste	3	5.08E−04	PIP/AZGP1/LPO
BP	GO:0050909	Sensory perception of taste	3	1.12E−03	PIP/AZGP1/LPO
BP	GO:0001895	Retina homeostasis	3	1.20E−03	PIP/AZGP1/PRR4
BP	GO:0001894	Tissue homeostasis	3	2.07E−02	PIP/AZGP1/PRR4
CC	GO:0032982	Myosin filament	2	5.69E−03	ACTG2/MYH11
CC	GO:0016459	Myosin complex	2	2.51E−02	ACTG2/MYH11

**Table 6:** KEGG pathways analysis results of DEGs.

Pathway ID	Description	Count	*p*-value	Genes
hsa04970	Salivary secretion	5	3.16E − 06	CST1/CST2/STATH/MUC7/LPO
hsa04061	Viral protein interaction with cytokine and cytokine receptor	3	2.07E − 03	CCL18/IL2RA/CCL13
hsa04060	Cytokine–cytokine receptor interaction	4	6.33E − 03	CCL18/IL2RA/CCL13/IL2RA
hsa04613	Neutrophil extracellular trap formation	3	1.24E − 02	AQP9/CR1/NCF2
hsa05140	Leishmaniasis	2	1.67E − 02	CR1/NCF2
hsa05208	Chemical carcinogenesis—reactive oxygen species	3	1.90E − 02	NCF2/GSTA2/LPO
hsa04610	Complement and coagulation cascades	2	2.01E − 02	CR1/F13A1

### Identification of hub DEGs genes by PPI network analysis

**Figure 5:**
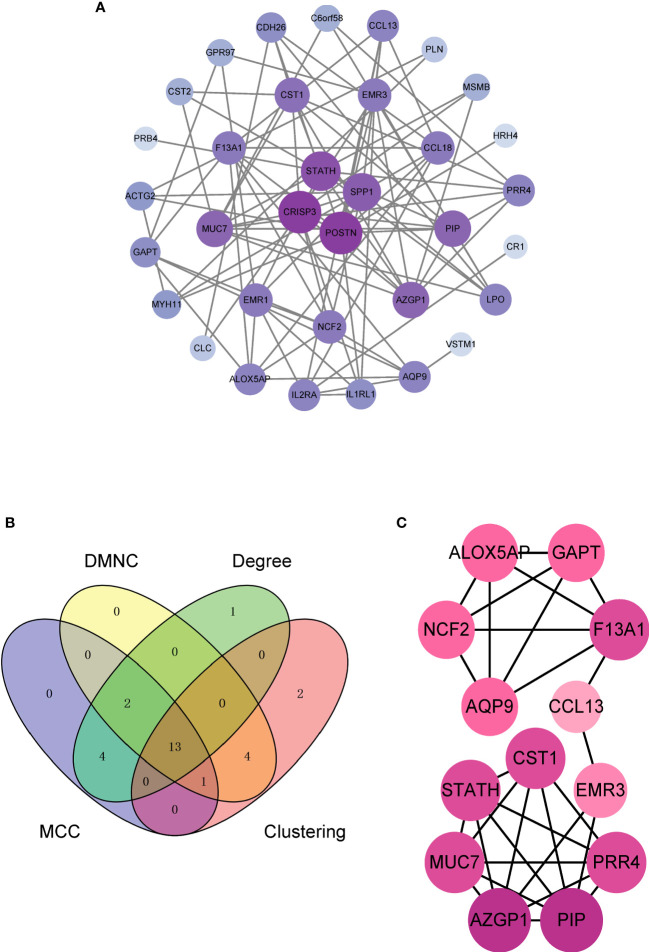
**(A)** PPI network of DEGs. **(B)** The Venn diagram of 13 hub genes obtained by integrating the four methods, **(C)** Subnetwork of 13 hub genes in the PPI network. The depth of the node color reflects the degree of connection (darker colors represent higher degrees; lighter colors represent lower degrees). PPI, protein–protein interaction; DEG, differentially expressed gene.

The PPI network constructed from the 36 DEGs in ACRSwNP was used to further explore the biological functions of DEGs (**Figure 5A**). The PPI network comprised 34 nodes and 98 edges, and isolated genes were deleted. Hub genes were identified by analyzing the PPI network using cytoHubba plugin in Cytoscape. The results from four cytohubba algorithms (including Degree, MCC, DMNC and Clustering Coefficient) were combined to identify 13 hub genes (**Figure 5B**). A total of 13 hub genes, namely, CST1, ALOX5AP, NCF2, GAPT, F13A1, CCL13, EMR3, PRR4, PIP, AZGP1, MUC7, STATH, and AQP9, were identified, which formed important nodes in the PPI network. This finding indicates that these 13 genes play key roles in development of ACRSwNP (**Figure 5C**).

### Target gene–miRNA network and the target gene–TF network

**Figure 6:**
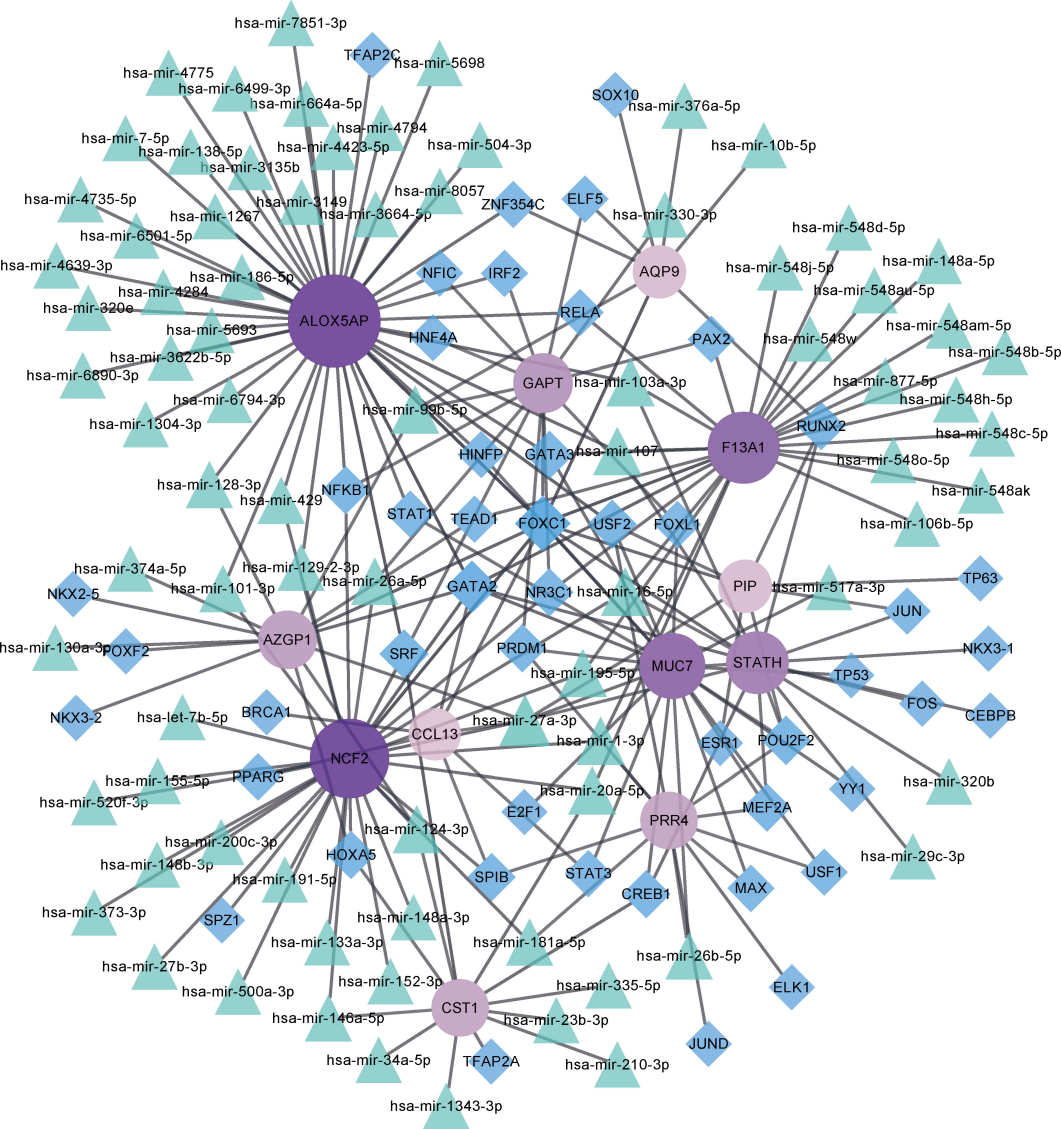
Target gene–miRNA and target gene–transcription factor (TF) networks. Purple circled nodes are genes, green triangle nodes are miRNAs, and blue diamond nodes represent TFs. Darker colors represent more connection points.

Key TF and miRNA involved in the process of disease development and progression at the post-transcriptional level were explored by constructing the target gene–miRNA network and the target gene–TF network (**Figure 6**). The top three targeted genes for miRNAs were ALOX5AP, NCF2, and F13A1, which were modulated by 32, 24, and 19 miRNAs, respectively. hsa-mir-27a-3p was identified as the miRNA that regulated the highest number of DEGs, regulating a total of six DEGs. The top three DEGs regulated by the highest number of TFs were MUC7, ALOX5AP, and GAPT, which were regulated by 17, 14, and 11 TFs, respectively. The TF that controlled the highest number of DEGs was FOXC1, which regulated a total of 10 genes.

### Key genes related to CRSwNP and asthma in ACRSwNP

**Figure 7:**
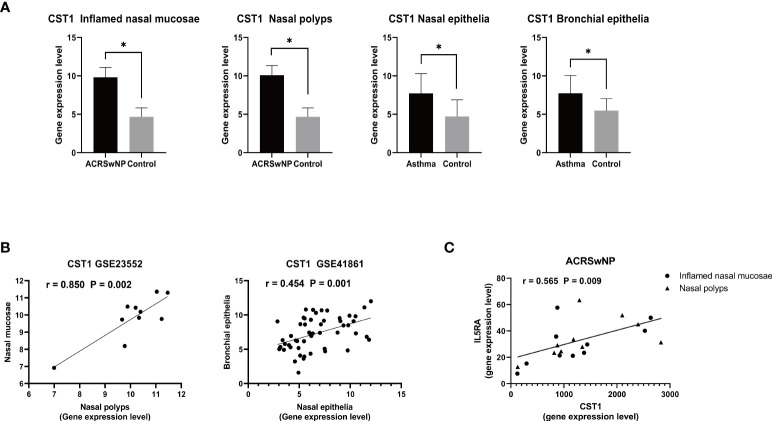
**(A)** Expression levels of CST1 in inflamed nasal mucosa and nasal polyps in ACRSwNP, and in the nasal epithelia and bronchial epithelia of asthmatics. *p < 0.05. **(B)** Scatter plot of CST1 expression levels in inflamed nasal mucosa and nasal polyps in ACRSwNP, and scatter plot of CST1 expression levels in the nasal epithelium and bronchial epithelium. **(C)** Correlation analysis and scatter plot of CST1 and IL5RA expression levels in inflamed nasal mucosa and nasal polyps in ACRSwNP.

In the dataset GSE41861, two DEGs were screened to meet the conditions, CST1 (adjusted *p*-value = 0.005081 and logFC = 3.003986) and CPA3 (adjusted *p*-value = 0.00000175 and logFC = 3.8023921). Analysis of DEGs obtained from the GSE41861 and GSE23552 datasets showed that CST1 was the only gene co-expressed in the nasal mucosa of asthma and ACRSwNP patients; that is, compared with normal controls, CST1 was highly expressed not only in the inflamed mucosa and nasal polyps of ACRSwNP patients, but also in the nasal mucosa of asthmatic patients. Furthermore, a search of the GSE41861 expression matrix revealed that CST1 was also upregulated in the bronchial epithelium of asthma patients compared to normal controls (**Figure 7A**). Correlation analysis showed that the expression levels of CST1 in nasal mucosa and nasal polyps of ACRSwNP patients were positively correlated (*r* = 0.850, *p* = 0.002), and the expression levels of CST1 in the nasal epithelium and bronchial epithelium were also positively correlated (*r* = 0.454, *p* = 0.001) (**Figure 7B**). These results suggest that CST1 may be a key gene that contributes to the severe symptoms and higher recurrence rate of ACRSwNP compared with CRSwNP alone. Moreover, we also analyzed the association between CST1 and the key cytokines and their receptors in type 2 inflammation, which showed that the expression levels of CST1 and IL5RA in patients with ACRSwNP were positively correlated (*r* = 0.565, *p* = 0.009), indicating that the upregulation of CST1 may enhance the type 2 inflammatory response of ACRSwNP (**Figure 7C**).

### Validation of hub genes

**Figure 8:**
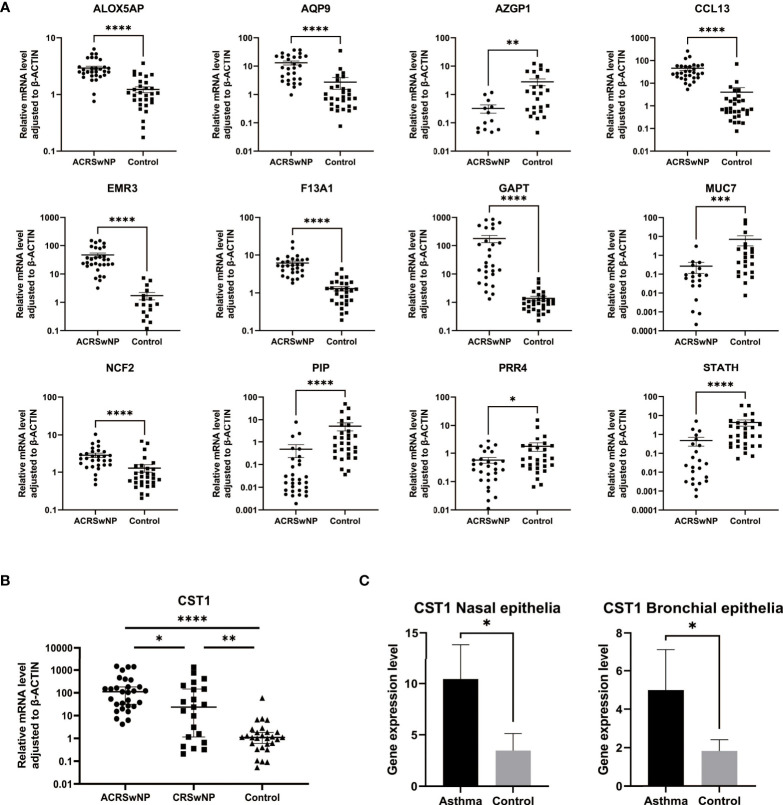
**(A)** RNA expression of *ALOX5AP, AQP9, AZGP1, CCL13, EMR3, F13A1, GAPT, MUC7, NCF2, PIP, PRR4*, and *STATH* in ACRSwNP and healthy control samples quantified using qRT-PCR, *p < 0.05; **p < 0.01; ***p < 0.001; ****p < 0.0001. **(B)** RNA expression of *CST1* in ACRSwNP, CRSwNP, and healthy control samples quantified using qRT-PCR. **(C)** In the dataset GSE104468, the RNA expression levels of CST1 in the nasal mucosal epithelium and bronchial epithelium of asthma patients.

Gene expression levels of 12 hub genes (ALOX5AP, AQP9, AZGP1, CCL13, EMR3, F13A1, GAPT, MUC7, NCF2, PIP, PRR4, and STATH) were validated by qRT-PCR using nasal polyps from ACRSwNP patients and nasal mucosa from healthy controls (**Figure 8A**). Expression levels of ALOX5AP, AQP9, CCL13, EMR3, F13A1, GAPT, and NCF2 were significantly higher in nasal polyp samples from ACRSwNP patients compared with the expression levels in samples from healthy subjects. In addition, expressions of AZGP1, STATH, PIP, MUC7, and PRR4 were significantly downregulated in ACRSwNP patients compared with healthy controls. The experimental results were consistent with the bioinformatics analysis results using GSE23552 microarray data. Furthermore, the expression level of CST1 in ACRSwNP, CRSwNP, and healthy controls was evaluated by qRT-PCR. The results showed that the expression level of CST1 was significantly higher in CRSwNP subjects compared with health controls (**Figure 8B**). Notably, the expression level of CST1 was significantly higher in ACRSwNP relative to the expression levels in CRSwNP subjects, and the difference was statistically significant. In addition, by retrieving the expression of CST1 in the dataset GSE104468, it was verified that CST1 was upregulated in the nasal mucosa and bronchial epithelium of asthma patients (**Figure 8C**).

### Screening candidate small-molecule drugs

The DEGs and their expression levels were imported into the CMap database for the prediction of potential small-molecule compounds or drugs that can reverse the altered expression of DEGs in ACRSwNP. A total of 10 negatively correlated small-molecule compounds with the highest enrichment scores were identified using the CMap database according to a criterion of *p*-value <0.05. These small-molecule compounds included STOCK1N-35874, profenamine, spaglumic acid, ethionamide, tacrolimus, cefamandole, calcium folinate, doxycycline, fluorocurarine, and butacaine, which are potential drugs for the treatment of ACRSwNP (**Table 7**).

**Table 7:** Ten chemicals predicted using the CMap database as potential treatments for ACRSwNP.

Rank	CMap name	CID	Molecular formula	Enrichment	*p*-value
1	STOCK1N-35874	-	-	−0.955	0.00427
2	Profenamine	3290	C19H24N2S	−0.855	0.00082
3	Spaglumic acid	188803	C11H16N2O8	−0.847	0.04688
4	Ethionamide	2761171	C8H10N2S	−0.814	0.01274
5	Tacrolimus	445643	C44H69NO12	−0.739	0.03656
6	Cefamandole	456255	C18H18N6O5S2	−0.738	0.00937
7	Calcium folinate	135403647	C20H21CaN7O7	−0.711	0.00429
8	Doxycycline	54671203	C22H24N2O8	−0.674	0.00867
9	Fluorocurarine	21586665	C20H23N2O+	−0.672	0.02594
10	Butacaine	2480	C18H30N2O2	−0.652	0.03467

CMap, Connectivity Map; CID, Compound ID. Notes: The molecular formula and CID of STOCK1N-35874 are not available on the PubChem database.

## Discussion

Several epidemiological studies report that CRSwNP is associated with clinical manifestations and pathophysiological mechanisms of asthma (27). Studies report IgE-mediated release of immune mediators and upregulation of type 2 cytokines in the upper and lower airways of patients with CRSwNP and asthma comorbidity (28). In addition, CRSwNP patients present with upregulation of genes associated with type 2 inflammation in the nasal mucosa which is significantly higher in patients with asthma (29). The common pathophysiology of the upper and lower airways has important implications for the diagnosis and treatment of respiratory comorbidities. However, in clinical practice, the nose and lungs are often treated as separate entities. Previous studies on CRSwNP mainly focused on the nasal polyp tissue of the upper airway. In this study, inflamed nasal mucosal tissue and nasal polyps were used to explore DEGs. In addition, we combined the upper and lower airway transcriptome data of asthma patients for the analysis. DEGs identified by this approach may play a more critical role in the pathogenesis of ACRSwNP. A total of 36 DEGs (21 upregulated and 15 downregulated) were identified from the GSE23552 dataset. A PPI network was constructed to explore associations among 36 genes, and 4 algorithms were used to identify 13 central genes at the protein expression level. Combined with the analysis results of GSE41861, we found that CST1 is highly expressed not only in the inflamed nasal mucosa and nasal polyps in ACRSwNP, but also in the nasal mucosa and bronchial epithelium of asthma patients. The expression level of CST1 in ACRSwNP was positively correlated with the mRNA expression level of IL5RA, a key cytokine of type 2 inflammation. qRT-PCR was conducted to verify the bioinformatics results, indicating that these genes are implicated in occurrence and development of ACRSwNP.

ALOX5AP encodes 5-lipoxygenase protein involved in leukotriene (LT) synthesis. LTs are important immunomodulatory and proinflammatory lipid mediators that modulate inflammatory responses by inducing leukocyte activation and chemotaxis in response to various immune stimuli (30, 31). Genome-wide gene and methylation expression profiles of nasal polyps in aspirin-intolerant asthma patients showed upregulation of ALOX5AP gene expression in nasal polyps whereas its methylation level was decreased (32). Blocking ALOX5AP can be an effective pharmacological approach to inhibit inflammation since LTs play a key role in inducing local inflammation in CRSwNP patients (33). However, further experiments should be conducted to verify these findings.

CCL13 gene encodes a CC family chemokine. CCL13 is involved in selective recruitment of cell lineages to inflamed tissues and their subsequent activation, thus mediating the progression of various inflammatory diseases (34). Previous studies report the overexpression of CCL13 in the bronchial epithelium and submucosa and in the bronchoalveolar lavage fluid obtained from asthmatic patients (35, 36). A previous study reported that although glucocorticoids can effectively suppress eosinophilic inflammation in nasal polyps, they do not significantly alter the expression level of CCL13 (37). This implies that CCL13 plays a key role in the pathogenesis of ACRSwNP. Blocking expression of CCL13 is a potential strategy for the treatment of ACRSwNP.

EMR3 gene encodes an epidermal growth factor, which belongs to the adhesion G protein-coupled receptor sub-family. The epidermal growth factor encoded by EMR3 is a member of the G protein-coupled receptor family, which also includes CD97, EMR1, EMR2, and EMR4 proteins. Previous studies report increased transcription of the G protein-coupled receptors EMR1 and EMR3 in polyp tissues of CRSwNP patients. The findings indicate that these proteins are involved in eosinophil adhesion and activation, which mediate the function of eosinophils in promoting progression of the disease (38). This implies that EMR3 is involved in the formation and development of nasal polyps by inducing eosinophil recruitment.

The NCF2 gene encodes neutrophil cytoplasmic factor 2, a subunit of NADPH oxidase involved in the mediation of innate immune responses (39). A study by Kaili et al. reported that NCF2 mRNA and protein levels are upregulated in nasal polyp tissues. This finding indicates that NCF2 plays a role in the pathogenesis of CRSwNP. Expression of NCF2 in different inflammatory cells found in nasal polyp tissues was explored using immunofluorescence staining. The results showed that NCF2 was expressed in eosinophils and neutrophils, but not in macrophages (40). NCF2 expression is significantly upregulated in CRSwNP patients compared with normal controls, and these patients test positive for *Staphylococcus aureus* biofilm (41). Inhibiting expression of NCF2 can be an effective pharmacological approach to abrogate formation of bacterial biofilm and mucosal chronic inflammation.

The AQP9 gene encodes Aquaporin 9, a member of the AQP water channel family that comprises aqueous glycerol channel proteins. These proteins are involved in the transport of water, glycerol, and lactic acid (42, 43). Studies have not explored the role of AQP9 in CRSwNP. However, previous studies report that AQP9 is involved in inflammatory diseases such as ulcerative colitis, systemic inflammatory response syndrome, and rheumatoid and osteoarthritis (44–46). Chemical inhibition of AQP9 in mouse models reduces the release of proinflammatory cytokines and chemokines, including IL-1α, IL-1β, IL-6, and IL-12, and TNF-α (47). Therefore, blocking AQP9 is a potential effective pharmacological approach for the abrogation of chronic inflammation and polyp growth.

The F13A1 gene encodes FXIII-A protein. Tetsuji et al. reported the upregulation of FXIII-A mRNA and protein expression in nasal polyps. Immunofluorescence analysis indicated that M2 macrophages were the predominant FXIIIA-producing cells in nasal polyps. Overproduction of FXIII-A by M2 macrophages can lead to excessive deposition of fibrin in the submucosa of nasal polyps, resulting in tissue remodeling and pathogenesis of CRSwNP (48). Therefore, inhibition of excess production of FXIII-A by M2 macrophages is a potential novel therapeutic target for reversing polyp tissue remodeling.

Lacrimal proline-rich 4 (PRR4) is a protein synthesized in acinar cells (49). Studies report that PRR4 is overexpressed in the submucosal glands of the human airway. It plays a role in the secretion of antimicrobial molecules and airway surface fluids from submucosal glands (50). Casado et al. identified PRR4 in the sputum of non-smokers and reported that PRR4 expression was downregulated in healthy smokers, whereas PRR4 was absent in the sputum of subjects in the chronic obstructive pulmonary disease (COPD) group (51). These findings indicate that downregulation of PRR4 may affect the physiological function of submucosal glands, leading to pathological changes in the airways, which may be the underlying pathogenesis of ACRSwNP.

The PIP gene encodes a single polypeptide chain named gross cystic fluid protein 15, which is secreted by various apocrine glands, including seminal vesicles, salivary glands, and sweat glands. Its abundance in mucosal tissues suggests a role in mucosal immunity (52). Our study results stand in contrast with those of Liu et al. who reported overexpression of PIP in microarray analysis of nasal polyps (53). A possible explanation of these discrepancies is that all of the subjects studied by Liu et al. received intranasal steroids for 1 month or more prior to surgery, which may influence expression levels. A recent study showed that PIP is related to the CRSwNP phenotype by weighted gene co-expression network analysis (54). Previous studies have speculated that the synthesis disorder of PIP may lead to the occurrence of nasolacrimal duct obstruction by making the mucosa of lacrimal drainage system susceptible to infection and inflammation (55). These findings indicate that the decreased expression of PIP may lead to CRSwNP by affecting the immune function of the nasal mucosa.

Zinc-α2-glycoprotein (ZAG) is a protein encoded by the AZGP1 gene (56). Studies report that ZAG affects sperm motility by regulating semen viscosity (57). In addition, ZAG has RNase activity; therefore, it may play a role in clearing RNA viruses (58). Even though a recent study reported that asthma and allergic rhinitis may not suffer from heavy virus load (59), CRSwNP is more susceptible to virus. These findings imply that the low expression of AZGP1 in nasal polyps may be implicated in the pathogenesis of ACRSwNP by affecting the biological function of mucus secreted and virus clearance. However, further experiments should be conducted to verify this postulation.

MUC7 encodes a small salivary mucin protein that protects T cells and epithelial cells from infection by binding antimicrobial proteins to retain them in the oral cavity (60). Studies report that mucins affect the rheological properties of mucus, leading to a series of pathophysiological changes including submucosal gland hyperplasia, increased number of airway goblet cells, and excessive mucin secretion, in patients with CRS. A study by Ali et al. showed that MUC7 is not expressed in normal sphenoid sinus mucosa, but is highly expressed mainly in the submucosal glands of the polyp tissue (61). However, Antón et al. did not detect MUC7 mucin in 26 samples of nasal polyps, while its expression was confirmed in 6 out of 11 normal mucosa samples, which was restricted to glands (62). The results of the present study are consistent with those of Antón et al. and opposite to the findings of Ali et al. This may be explained by the possibility that the polypoid middle turbinate contains serous mucous glands as reported by Ali et al., which are not found in normal nasal polyps (63).

STATH encodes statherin protein, which is a peptide with antimicrobial properties mainly expressed in the saliva, upper airways, and nasal secretions (64). Liu et al. used DNA microarray technology for the analysis of nasal polyps in CRSwNP patients treated with intranasal glucocorticoids and demonstrated that the expression level of STATH was higher in CRSwNP patients compared with that of normal turbinate mucosa (53). This is contrary to our findings, which may indicate that glucocorticoids can reverse the downregulated expression of STATH in nasal polyp. Statherin has epitopes that promote the growth and adhesion of certain microorganisms in the oral cavity (65), suggesting that decreased expression of STATH may cause dysbiosis of nasal mucosa and participate in the pathogenesis of ACRSwNP. In addition, it is noteworthy that similar to STATH, the proteins encoded by PIP, MUC7, AZGP1, and PRR4 in the central gene are all related to the antimicrobial defense of mucosa, and all are downregulated in ACRSwNP. It has been demonstrated that viral infection releases TSLP and IL-25 from nasal polyp epithelial cells to help maintain and amplify the T2 immune response common in CRSwNP (66), and it is reasonable to speculate that downregulation of these genes could affect the function of the nasal mucosal epithelial barrier, increases the risk of viral infection, and participates in the pathogenic mechanism of CRSwNP through the aforementioned mechanisms.

The CST1 gene encodes Cystatin SN protein. Cystatin SN is a member of the type 2 cystatin protein superfamily. A study by Yan et al. reported that CST1 expression in nasal polyps was significantly upregulated in eosinophilic CRSwNP (ECRSwNP) patients but downregulated in noneosinophilic CRSwNP (nonECRSwNP) patients compared with the levels in healthy controls. CST1 expression was further upregulated in ECRSwNP patients with asthma comorbidity and the expression level was correlated with the percentage of eosinophils in tissue samples (67). The results of the present study showed that CST1 expression was upregulated in pure CRSwNP samples compared with the expression in controls. In addition, the expression level of CST1 was higher in the ACRSwNP group, which was statistically significant compared with the CRSwNP group. These findings were consistent with the study reported by Yan et al. Ulrika et al. demonstrated that nasal CST1 expression is associated with clinical and biochemical markers for asthma and airway allergy. A recent study explored the relationship between COPD and blood eosinophil count and airway epithelial transcriptome, showing that CST1 was the only gene positively associated with blood eosinophil count in asthma and COPD patients (68). CST1 is highly expressed in the airway epithelium of Th2-high asthma patients, and is one of the biomarkers to distinguish Th2-high from Th2-low asthma (69). These findings indicate that CST1 plays a key role in the pathogenesis of asthma and CRSwNP, and is a key biomarker linking CRSwNP and asthma.

More than 90% of nonubiquitous transcripts expressed in the bronchial airways are also expressed in the nasal airways, with strong correlation between expression of the nasal and bronchial airways transcriptomes (70). Multiple studies have shown that asthma can alter gene expression levels in the nasal mucosa of patients, and nasal transcriptomics can provide important insights into asthma (71, 72). However, there is still a lack of research on whether there is a link between asthma-induced changes in the nasal mucosa transcriptome and CRSwNP, and whether it can cause changes in CRSwNP gene expression levels. In our study, by analyzing different datasets and performing qRT-PCR experiments, we found that compared with normal controls, CST1 was highly expressed in the inflamed nasal mucosa and nasal polyps in ACRSwNP patients, highly expressed in nasal polyps in CRSwNP patients (but less than the ACRSwNP group), and highly expressed in the nasal mucosa and bronchial epithelium of asthma patients. In addition, the expression levels of CST1 in the nasal mucosa and nasal polyps of ACRSwNP patients were positively correlated (*r* = 0.850, *p* = 0.002), and the expression levels in the nasal epithelium and bronchial epithelium were also positively correlated (*r* = 0.454, *p* = 0.001). These findings support the united airways concept. Based on these findings, we speculated that asthma-induced changes in upper airway gene expression levels lead to increased upper airway susceptibility to CRSwNP, and compared with patients without asthma, after the onset of CRSwNP, the expression of corresponding genes will be significantly upregulated in a superimposed manner, resulting in more severe upper airway inflammation. The hypothesis we put forward explains, to a certain extent, why asthma patients are prone to suffer from chronic sinusitis, and why patients with ACRSwNP have a more severe inflammatory state than patients with CRSwNP alone. Likewise, the epidemiological features of asthma onset mainly in childhood and ACRSwNP mainly in adulthood are also consistent with our hypothesis (28). In this study, we found that asthma participates in the pathogenic mechanism of ACRSwNP by upregulating the expression level of CST1 in the upper airway, and CST1 is associated with the expression level of the type 2 inflammatory cytokine receptor IL5RA. In view of our screening criteria (adjusted *p*-value < 0.05 and |logFC > 2|, it is reasonable to believe that the number of genes through which asthma can cause changes in expression levels in the upper airway and that are involved in CRSwNP is far more than one CST1, but CST1 may be the most significantly upregulated gene.

GO analysis results in the present study showed that upregulated genes were mainly enriched for immune cell proliferation, adaptive immune response, negative regulation of immune response, regulation of inflammatory response and cytokine activity. Dysregulation of these processes indicates that complex immune responses and chronic inflammation are implicated in the formation and development of nasal polyps, which differ with CRSwNP state (73). Furthermore, downregulated genes were predominantly enriched for gustatory sensory perception, tissue homeostasis, and muscle system process. This implies that dysregulation of these genes may be involved in mechanisms of olfactory dysfunction, disruption of the sinus epithelial cell barrier, and tissue remodeling in ACRSwNP. KEGG pathway analysis showed that DEGs were mainly related to enrichment of salivary secretion and interaction of viral proteins with cytokines and cytokine receptors. Notably, several genes including CST1, CST2, STATH, MUC7, and LPO were significantly associated with enrichment of the salivary secretion pathway. This finding indicates that dysregulation of the salivary secretion pathway in CRSwNP may lead to major defects in innate defense responses, ultimately increasing susceptibility to microbial colonization and promoting development of sinus mucosa persistent inflammation (74). The enriched pathways are key biological processes leading to pathogen invasion, immune regulation disturbances, and chronic inflammatory responses, as well as tissue remodeling. Therefore, they ultimately lead to severe immune responses and formation of nasal polyps.

The top three targeted DEGs in the miRNA gene network were ALOX5AP, NCF2, and F13A1. The relationship between ALOX5AP, NCF2, F13A1, and ACRSwNP has been explored. In the present study, hsa-mir-27a-3p modulated the highest number of DEGs. hsa-mir-27a-3p may promote the occurrence of inflammation and the formation of nasal polyps by regulating the expression of AZGP1, NCF2, CCL13, MUC7, PIP, and STATH. The top three target genes in the TF gene network were MUC7, ALOX5AP, and GAPT. Studies have not fully explored the relationship between GAPT and ACRSwNP. FOXC1 transcription factor was associated with the highest number of DEGs. FOXC1 belongs to the forkhead transcription factor superfamily. Members of this family play an important role in life processes such as angiogenesis, early embryonic development, cell metabolism, and cell lifespan (75). Studies report that FOXC1 modulates occurrence, development, and metastasis of tumors (76). Moreover, FOXC1 is involved in the transcription of ALOX5AP, NCF2, and F13A1, implying that FOXC1 mediates progression of CRSwNP by controlling the transcription of ALOX5AP, NCF2, and F13A1.

The CMap database was used to identify potential drugs related to DEGs. The DEGs obtained by the expression profile chip were analyzed, and the top 10 candidate small molecules significantly negatively correlated with the differential gene expression profile of ACRSwNP were selected. These small molecules can inhibit the expression of DEGs and have the potential to treat ACRSwNP. STOCK1N-35874 is a cytotoxic quinoline alkaloid that inhibits the DNA enzyme topoisomerase, which is isolated from the bark and stem of *Camptotheca acuminata* (77). STOCK1N-35874 showed marked anticancer activity in preliminary clinical trials, and its analogs have been used in cancer chemotherapy (78). Profenamine, a phenothiazine derivative with anticholinergic, antihistaminergic, and antiadrenergic effects, is currently mainly used to treat Parkinson’s disease by improving muscle control and reducing stiffness (79). Spaglumic acid, a mast cell stabilizer that blocks the release of histamine and other mediators by inhibiting mast cell degranulation, is used to treat allergic conjunctivitis (80). Ethionamide, a nicotinamide derivative with antibacterial activity, is an important second-line drug for the treatment of tuberculosis, often used in MDR-TB regimens (81). Tacrolimus is an immunosuppressive drug whose main use is after organ transplant to reduce the activity of the patient’s immune system and, thus, the risk of organ rejection (82). Cefamandole is a second-generation cephalosporin antibiotic with bactericidal activity, belonging to the β-lactam antibiotics, which is used to treat various infections caused by sensitive bacteria, such as lower respiratory infections, urinary tract infections, skin infections, and bone and joint infections (83). Calcium folinate is a folate analog used to treat the toxic effects of methotrexate and other folate antagonists, to treat megaloblastic anemia, and to provide palliative treatment of colorectal cancer (84). Doxycycline is a semi-synthetic tetracycline antibiotic used to inhibit bacterial protein synthesis. It has antibacterial, antimalarial, geroprotector, anti-inflammatory, and immunomodulatory effects for the treatment of non-gonococcal urethritis and cervicitis, exacerbation of bronchitis in patients with COPD, and periodontitis in adults (85). Fluorocurarine chloride is a short-acting selective sympathetic ganglioblocker. Butacaine is a reversible nerve conduction blocker that acts on the nervous system and nerve fibers, causing both sensory and motor paralysis. These drugs have not yet been reported as therapies for ACRSwNP. This work may help to provide new strategies for the drug treatment of ACRSwNP.

The present study has some limitations. First, the small sample size used for bioinformatics analysis may affect the reliability of the results. Second, the diagnosis of asthma in the recruited ACRSwNP patients was based on the past medical records, rather than objective examination when they were admitted to our department, and the current asthma status of the patients could not be determined. Third, validation was only performed at the expression level of the genes, whereas the potential mechanisms of these genes in ACRSwNP were not explored. Fourth, due to the lack of patient data in GSE41861 and GSE104468, we do not know whether the patients in these datasets have other respiratory diseases such as chronic sinusitis.

Gene expression profiles of the GEO dataset were evaluated using multiple bioinformatics tools, and 13 potentially hub genes and signaling pathways associated with the pathogenesis of ACRSwNP were identified. In addition, potential drug compounds for ACRSwNP were predicted. Furthermore, our findings found that asthma may be involved in the pathogenesis of ACRSwNP by altering the expression levels of upper airway genes, of which CST1 is one of the key genes. However, further prospective clinical trials should be conducted to verify these findings.

## Data availability statement

The datasets presented in this study can be found in online repositories. The names of the repository/repositories and accession number(s) can be found below: https://www.ncbi.nlm.nih.gov/geo/, GSE23552 https://www.ncbi.nlm.nih.gov/geo/, GSE104468 https://www.ncbi.nlm.nih.gov/geo/, GSE41861.

## Ethics statement

The studies involving human participants were reviewed and approved by The Medical Ethics Committee of Qilu Hospital of Shandong University. Written informed consent to participate in this study was provided by the participants or the participants’ legal guardian/next of kin.

## Author contributions

XL and XF designed the study. MW drafted the manuscript. MW, ST, XY, XX, YL, and SH contributed to the enrollment of subjects and data collection. MW, ST, XY, and XX performed the experiments. MW performed the analysis. XF and XL contributed to the interpretation of the results, and reviewed and edited the manuscript. All authors contributed to the article and approved the submitted version.

## Funding

This research is supported by the National Natural Science Foundation of China (82171106 and 81700890), the Taishan Scholar Program of Shandong Province (tsqn202103166), the China Postdoctoral Science Foundation (2021M691938), and the Shandong Natural Science Foundation (ZR2021MH117).

## Conflict of interest

The authors declare that the research was conducted in the absence of any commercial or financial relationships that could be construed as a potential conflict of interest.

## Publisher’s note

All claims expressed in this article are solely those of the authors and do not necessarily represent those of their affiliated organizations, or those of the publisher, the editors and the reviewers. Any product that may be evaluated in this article, or claim that may be made by its manufacturer, is not guaranteed or endorsed by the publisher.
